# Ant genus *Strongylognathus* (Hymenoptera, Formicidae) in Bulgaria: a preliminary review

**DOI:** 10.3897/BDJ.9.e65742

**Published:** 2021-05-21

**Authors:** Albena Lapeva-Gjonova, Alexander G. Radchenko

**Affiliations:** 1 Sofia University, Sofia, Bulgaria Sofia University Sofia Bulgaria; 2 Schmalhausen Institute of Zoology of the National Academy of Sciences of Ukraine, Kiev, Ukraine Schmalhausen Institute of Zoology of the National Academy of Sciences of Ukraine Kiev Ukraine

**Keywords:** ants, Balkans, fauna, social parasites, taxonomy, *Strongylognathus karawajewi*, *S. huberi dalmaticus*, *S. afer*, *S. italicus*, new records, key

## Abstract

**Background:**

*Strongylognathus* Mayr, 1853 is a Palaearctic genus, comprising 25 ant species and one subspecies, all permanent social parasites, infesting colonies of various species of *Tetramorium* Mayr, 1855. They have patchy distribution throughout their areas and most of them are very rare and listed as vulnerable.

The taxonomy of the *Strongylognathus huberi* group needs thorough revision and the results presented below can be considered as preliminary.

**New information:**

Four species of the socially parasitic ant genus *Strongylognathus* (*S. karawajewi* Pisarski, 1966, *S. huberi dalmaticus* Baroni Urbani, 1969, *S. afer* Emery, 1884 and *S. italicus* Finzi, 1924) are recorded for the first time from Bulgaria and, together with the previously-known *S. testaceus* and *S. bulgaricus* stat. rev., their total number reaches six. The taxonomic position and geographic distribution of all species are discussed and a Key for their identification, based on worker caste, is compiled.

## Introduction

*Strongylognathus* Mayr, 1853 is a Palaearctic genus, comprising 25 ant species and one subspecies distributed from north-west Africa to Japan, but with a gap in east Siberia, Mongolia and Russian Far East (Radchenko et al. 2017, Bolton 2021). It differs significantly from other ants of the subfamily Myrmicinae by its falcate, toothless mandibles without a defined masticatory margin.

*Strongylognathus* is divided into two species-groups: the *testaceus*- and *huberi*-group (Bolton 1976). The species of the first group have a strongly concave occipital margin and prominent posterio-lateral corners of the head, while the species of the *huberi*-group have straight or, at most, a very shallowly concave occipital margin and rounded, not prominent, posterio-lateral corners of the head.

The *testaceus*-species group contains four species – *S. testaceus* (Schenck, 1852), *S. karawajewi* Pisarski, 1966, *S. potanini* Radchenko, 1995 and *S. tylonus* Wei, Xu & He, 2001. The first two species are distributed in the West Palaearctic from Atlantic coasts to middle Asia and west Siberia, but the latter ones are known from China (Radchenko et al. 2017).

The majority of the members from the *huberi*-group (19 species and one subspecies) are distributed in the Mediterranean Region, Southern and Central Europe, south of east Europe, Caucasus, Anatolia, Near and Middle East, Turkmenistan, Iran and Afghanistan and only two species are known from China, Korea and Japan (Radchenko 2005, Radchenko et al. 2017). In general, *Strongylognathus* species have patchy distribution throughout their areas and most of them are listed as vulnerable (IUCN 2021).

All *Strongylognathus* species are permanent social parasites, infesting colonies of various species of *Tetramorium* Mayr, 1855. Many species of the *huberi*-group are dulotic, engage slave raids and attack colonies of *Tetramorium*, retrieving their brood. In contrast, species of the *testaceus* species-group appear to be queen-tolerant parasites and do not engage in slave raids and co-exist with the host queen, which produces only worker caste (Buschinger 2009, Sanetra and Buschinger 2000, D'Ettorre and Heinze 2001).

So far, two *Strongylognathus* species have been recorded from Bulgaria – *S. testaceus* and *S. bulgaricus* Pisarski, 1966 (Viehmeyer 1922, Atanassov 1964, Pisarski 1966, Atanassov and Vasileva 1976, Atanassov and Dlussky 1992, Lapeva-Gjonova 2004, Lapeva-Gjonova et al. 2010, Lapeva-Gjonova and Kiran 2012).

Below, we record, for the first time, four *Strongylognathus* species from Bulgaria: *S. karawajewi*, *S. huberi dalmaticus* Baroni Urbani, 1969, *S. afer* Emery, 1884 and *S. italicus* Finzi, 1924. Thereby, the currently-known *Strongylognathus* species to the country has increased to six. Their taxonomic position and a Key for the identification of Bulgarian *Strongylognathus* species, based on the worker caste, are provided.

The taxonomy of the *Strongylognathus huberi* group needs thorough revision and it is simply impossible to unambiguously identify most of the West Palaearctic species and the results presented below can be considered as preliminary.

## Materials and methods

**Figure 1.**
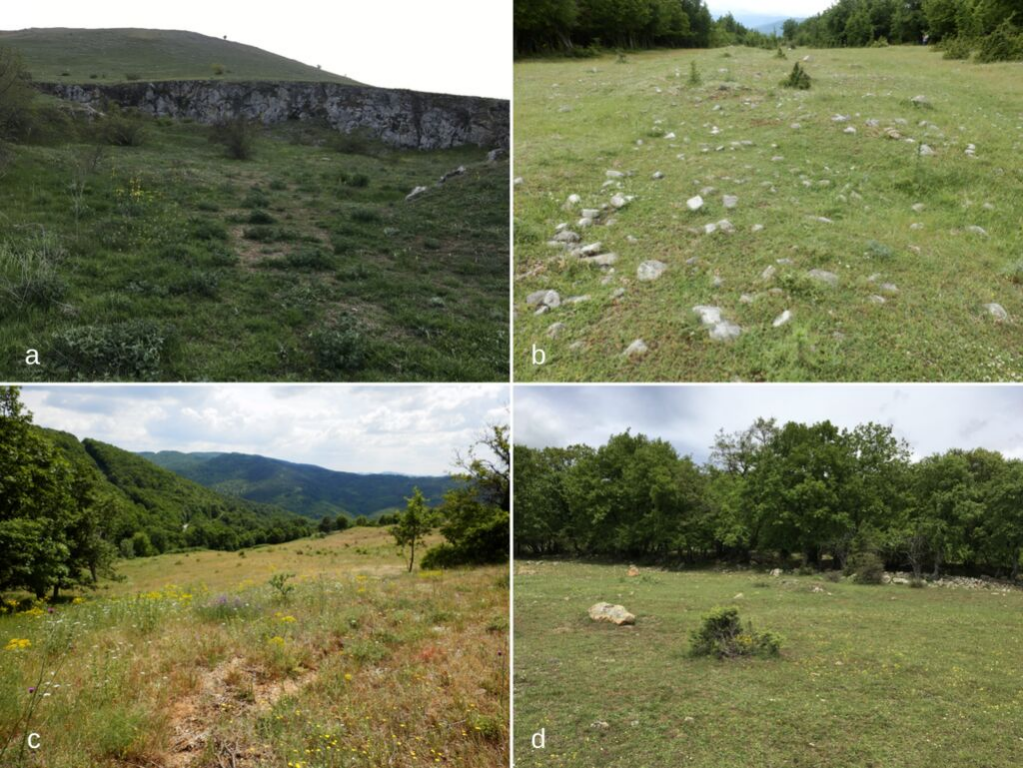
Photos of some collection sites: a – Thracian plain, Besaparski hills, near Ognyanovo vill. (habitat of *Strongylognathus karawajewi*); b – Stara planina, near Karnare vill. (habitat of *S. karawajewi*); c – Eastern Rhodopes, near Chernichino vill. (habitat of *S. karawajewi* and *S. italicus*); d – Eastern Rhodopes, near Gaberovo vill. (habitat of *S. karawajewi* and *S. afer*).

*Strongylognathus* species were collected during a myrmecological survey in Bulgaria by the first co-author of the paper (ALG). In recent years, special attention has been paid to the southern regions of the country (Eastern Rhodopes, Thracian plain, Strandzha, Slavyanka and Maleshevska Mountains), where the ant fauna is most diverse, but has not been properly investigated yet in detail. Collected material is preserved at the Biological Faculty, University of Sofia, Bulgaria (BFUS). The examined type specimens of *S. karawajewi* Pisarski, 1966 are preserved in the collections of the Schmalhausen Institute of Zoology NAS of Ukraine, Kiev (SIZK) and the Museum and Institute of Zoology PAS, Warsaw (MIIZ). All *Tetramorium* nests, infested by *Strongylognathus*, were located in the ground under stones. Photos of some collection sites are shown in Fig. 1. Since there are no modern keys for identification of the European *Strongylognathus*, we used data from various publications for their identification (Pisarski 1966, Baroni Urbani 1969, Radchenko 1985, Radchenko 1991, Dlussky et al. 1990, Sanetra et al. 1999, Sanetra and Güsten 2001, Schulz and Sanetra 2002, Borowiec and Salata 2013, Seifert 2018), comparative material from SIZK, MIIZ, Zoological Museum of the Moscow State University, Zoological Institute RAS, St-Petersburg and Petr Werner’s collection, Prague, as well as the original description of all taxa and images of the type and non-type specimens on the AntWeb (2021) website. The specimens for scanning electron microscopy (SEM) were gold-coated in a vacuum unit and then images were taken using the microscope LYRA/TESCAN 5007, operating at 10 kV.

Six measurements of specimens (accurate to 0.01 mm) were taken and used to calculate four indices:

- HL – maximum length of head in dorsal view, measured in a straight line from the most anterior point of clypeus to the posteriormost point of occipital margin;
- HW – maximum width of head in dorsal view behind (above) the eyes;
- SL – maximum straight-line length of scape from its articulation with the condylar bulb to the distal edge of the scape;
- ML – diagonal length of the mesosoma seen in profile, from the anterior end of the neck shield to the posterior margin of the propodeal lobes;
- PW – maximum width of the petiole from above;
- PPW – maximum width of the postpetiole from above.
- Indices: CI = HL/HW, SI = SL/HL, PI = PW/PPW, PPI = PPW/HW

## Taxon treatments

### Strongylognathus testaceus

(Schenck, 1852)

A69520F6-E614-5535-9381-98E1F875D23D

Eciton testaceum Schenck, 1852: 117, w, q, Germany.
Strongylognathus testaceus : Mayr 1853: 390, m; all subsequent authors.
Eciton testaceum Schenck, 1852: 117, w, q, Germany.
Strongylognathus testaceus : Mayr 1853: 390, m; all subsequent authors.

#### Materials

- **Type status:** Other material. **Occurrence:** recordedBy: A. Lapeva-Gjonova; individualCount: 1; sex: worker; **Taxon:** scientificName: *Strongylognathus testaceus*; order: Hymenoptera; family: Formicidae; genus: Strongylognathus; taxonRank: Species; **Location:** country: Bulgaria; stateProvince: Burgas; municipality: Malko Tarnovo; locality: Strandzha Mt., Bliznak vill.; minimumElevationInMeters: 335; locationRemarks: oak forest, in a nest of Tetramorium cf. caespitum; decimalLatitude: 42.195; decimalLongitude: 27.3305; **Event:** eventDate: 05-05-2009; **Record Level:** collectionID: BFUS; basisOfRecord: PreservedSpecimen
- **Type status:** Other material. **Occurrence:** recordedBy: A. Lapeva-Gjonova; individualCount: 1; sex: worker; **Taxon:** scientificName: *Strongylognathus testaceus*; order: Hymenoptera; family: Formicidae; genus: Strongylognathus; taxonRank: Species; **Location:** country: Bulgaria; stateProvince: Blagoevgrad; municipality: Sandanski; locality: Slavyanka Mt., near Goleshevo vill.; minimumElevationInMeters: 931; locationRemarks: rocky grassland with shrubs, in a nest of Tetramorium cf. caespitum; decimalLatitude: 41.4108; decimalLongitude: 23.5887; **Event:** eventDate: 04-05-2013; **Record Level:** collectionID: BFUS; basisOfRecord: PreservedSpecimen
- **Type status:** Other material. **Occurrence:** recordedBy: A. Lapeva-Gjonova; individualCount: 10; sex: workers; **Taxon:** scientificName: *Strongylognathus testaceus*; order: Hymenoptera; family: Formicidae; genus: Strongylognathus; taxonRank: Species; **Location:** country: Bulgaria; stateProvince: Blagoevgrad; municipality: Strumyani; locality: Maleshevska Mt., near Igralishte vill.; minimumElevationInMeters: 916; locationRemarks: along a road at the edge of an oak forest, in a nest of Tetramorium cf. caespitum; decimalLatitude: 41.5688; decimalLongitude: 23.1213; **Event:** eventDate: 17-08-2014; **Record Level:** collectionID: BFUS; basisOfRecord: PreservedSpecimen
- **Type status:** Other material. **Occurrence:** recordedBy: A. Lapeva-Gjonova; individualCount: 11; sex: workers; **Taxon:** scientificName: *Strongylognathus testaceus*; order: Hymenoptera; family: Formicidae; genus: Strongylognathus; taxonRank: Species; **Location:** country: Bulgaria; stateProvince: Lovech; municipality: Lukovit; locality: Western Predbalkan, near Karlukovo vill.; minimumElevationInMeters: 299; locationRemarks: rocky grassland, in nests of Tetramorium cf. caespitum; decimalLatitude: 43.1556; decimalLongitude: 24.0885; **Event:** eventDate: 05-06-2016; **Record Level:** collectionID: BFUS; basisOfRecord: PreservedSpecimen

#### Description

SEM images: Fig. 2

#### Taxon discussion

*Strongylognathus testaceus* appears to be the most common species of this genus, it widely spreading in Central and Southern Europe, southern part of east Europe, the Caucasus, Anatolia, south of west Siberia and northern Kazakhstan (Radchenko 1985, Radchenko 1991, Radchenko 2016, Schulz and Sanetra 2002). In Bulgaria, it has been found in the southern part of the country: in the southern Black Sea coast (Ahtopol), Strandzha Mt. (Bliznak vill.), the Kozhuh volcanic hill and Belasitsa Mountain (Lapeva-Gjonova et al. 2010, Lapeva-Gjonova and Kiran 2012). The previous record of *S. testaceus* from Eastern Rhodopes (Dedetz vill.) (Lapeva-Gjonova 2004) refers to *S. karawajewi* (misidentification).

*Strongylognathus testaceus* has long been known as a social parasite of *Tetramorium caespitum* (Linnaeus, 1758), but was recently found in the nests of *T. alpestre* Steiner, Schlick-Steiner & Seifert, 2010 and *T. impurum* (Foerster, 1850) (Wagner et al. 2017; our unpublished data).

### Strongylognathus karawajewi

Pisarski, 1966

F56AC838-0C5F-5313-B68E-9EA5136B5936

Strongylognathus karawajewi Pisarski, 1966: 521, w, Ukraine; Radchenko 1985: 1519, q; Radchenko 1991: 88, m; all subsequent authors.
Strongylognathus karawajewi Pisarski, 1966: 521, w, Ukraine; Radchenko 1985: 1519, q; Radchenko 1991: 88, m; all subsequent authors.

#### Materials

- **Type status:** Paratype. **Occurrence:** occurrenceRemarks: 3 workers on two pins, “???????, ????, ?. ????????” [Magartsch, Crimea, A. Riazantzev], “4790. Coll. Karavaievi”, “*Strongylognathus testaceus* Schenck Karawajew det.”, “*Strongylognathus karawajewi* sp. n. det. B. Pisarski”; **Taxon:** scientificName: *Strongylognathus karawajewi*; order: Hymenoptera; family: Formicidae; genus: Strongylognathus; taxonRank: Species; **Record Level:** collectionCode: SIZK; basisOfRecord: PreservedSpecimen
- **Type status:** Paratype. **Occurrence:** occurrenceRemarks: 2 workers, “Magartsch, Crimée, A. Riazantzev”, “*Strongylognathus karawajewi* sp. n. det. B. Pisarski”; **Taxon:** scientificName: *Strongylognathus karawajewi*; order: Hymenoptera; family: Formicidae; genus: Strongylognathus; taxonRank: Species; **Record Level:** collectionCode: MIIZ; basisOfRecord: PreservedSpecimen
- **Type status:** Paratype. **Occurrence:** occurrenceRemarks: 1 worker, "Krim, Magaratsch", "*Strongylognathus karawajewi* sp. n. det. B. Pisarski"; **Taxon:** scientificName: *Strongylognathus karawajewi*; order: Hymenoptera; family: Formicidae; genus: Strongylognathus; taxonRank: Species; **Record Level:** collectionCode: MIIZ; basisOfRecord: PreservedSpecimen
- **Type status:** Paratype. **Occurrence:** occurrenceRemarks: 1 worker, "Krim, Magaratsch, 23-III-1903, leg. Riazancev, Nr. 4790", "*Strongylognathus karawajewi* sp. n. det. B. Pisarski"; **Taxon:** scientificName: *Strongylognathus karawajewi*; order: Hymenoptera; family: Formicidae; genus: Strongylognathus; taxonRank: Species; **Record Level:** collectionCode: MIIZ; basisOfRecord: PreservedSpecimen
- **Type status:** Other material. **Occurrence:** recordedBy: A. Lapeva-Gjonova; individualCount: 8; sex: workers; **Taxon:** scientificName: *Strongylognathus karawajewi*; order: Hymenoptera; family: Formicidae; genus: Strongylognathus; taxonRank: Species; **Location:** country: Bulgaria; stateProvince: Kardzhali; municipality: Kirkovo; locality: Eastern Rhodopes, near Dedetz vill.; minimumElevationInMeters: 375; locationRemarks: in a nest of *Tetramorium hungaricum* Röszler, 1935; decimalLatitude: 41.38; decimalLongitude: 25.2233; **Event:** eventDate: 27-04-2003; **Record Level:** collectionCode: BFUS; basisOfRecord: PreservedSpecimen
- **Type status:** Other material. **Occurrence:** recordedBy: A. Lapeva-Gjonova; individualCount: 6; sex: workers; **Taxon:** scientificName: *Strongylognathus karawajewi*; order: Hymenoptera; family: Formicidae; genus: Strongylognathus; taxonRank: Species; **Location:** country: Bulgaria; stateProvince: Haskovo; municipality: Ivaylovgrad; locality: Eastern Rhodopes, near Meden buk vill.; minimumElevationInMeters: 124; locationRemarks: in a nest of *Tetramorium hungaricum* Röszler, 1935; decimalLatitude: 41.3822; decimalLongitude: 26.017; **Event:** eventDate: 04-05-2009; **Record Level:** collectionCode: BFUS; basisOfRecord: PreservedSpecimen
- **Type status:** Other material. **Occurrence:** recordedBy: A. Lapeva-Gjonova; individualCount: 5; sex: workers; **Taxon:** scientificName: *Strongylognathus karawajewi*; order: Hymenoptera; family: Formicidae; genus: Strongylognathus; taxonRank: Species; **Location:** country: Bulgaria; stateProvince: Haskovo; municipality: Ivaylovgrad; locality: Eastern Rhodopes, near Meden buk vill.; minimumElevationInMeters: 124; locationRemarks: in a nest of *Tetramorium hungaricum* Röszler, 1935; decimalLatitude: 41.3822; decimalLongitude: 26.017; **Event:** eventDate: 09-04-2013; **Record Level:** collectionCode: BFUS; basisOfRecord: PreservedSpecimen
- **Type status:** Other material. **Occurrence:** recordedBy: A. Lapeva-Gjonova; individualCount: 3; sex: workers; **Taxon:** scientificName: *Strongylognathus karawajewi*; order: Hymenoptera; family: Formicidae; genus: Strongylognathus; taxonRank: Species; **Location:** country: Bulgaria; stateProvince: Plovdiv; municipality: Karlovo; locality: Stara planina, near Karnare vill.; minimumElevationInMeters: 644; locationRemarks: in nests of Tetramorium cf. caespitum; decimalLatitude: 42.7168; decimalLongitude: 24.6343; **Event:** eventDate: 20-06-2014; **Record Level:** collectionCode: BFUS; basisOfRecord: PreservedSpecimen
- **Type status:** Other material. **Occurrence:** recordedBy: A. Lapeva-Gjonova; individualCount: 25; sex: queens; **Taxon:** scientificName: *Strongylognathus karawajewi*; order: Hymenoptera; family: Formicidae; genus: Strongylognathus; taxonRank: Species; **Location:** country: Bulgaria; stateProvince: Plovdiv; municipality: Karlovo; locality: Stara planina, near Karnare vill.; minimumElevationInMeters: 644; locationRemarks: in nests of Tetramorium cf. caespitum; decimalLatitude: 42.7168; decimalLongitude: 24.6343; **Event:** eventDate: 20-06-2014; **Record Level:** collectionCode: BFUS; basisOfRecord: PreservedSpecimen
- **Type status:** Other material. **Occurrence:** recordedBy: A. Lapeva-Gjonova; individualCount: 23; sex: males; **Taxon:** scientificName: *Strongylognathus karawajewi*; order: Hymenoptera; family: Formicidae; genus: Strongylognathus; taxonRank: Species; **Location:** country: Bulgaria; stateProvince: Plovdiv; municipality: Karlovo; locality: Stara planina, near Karnare vill.; minimumElevationInMeters: 644; locationRemarks: in nests of Tetramorium cf. caespitum; decimalLatitude: 42.7168; decimalLongitude: 24.6343; **Event:** eventDate: 20-06-2014; **Record Level:** collectionCode: BFUS; basisOfRecord: PreservedSpecimen
- **Type status:** Other material. **Occurrence:** recordedBy: A. Lapeva-Gjonova; individualCount: 4; sex: workers; **Taxon:** scientificName: *Strongylognathus karawajewi*; order: Hymenoptera; family: Formicidae; genus: Strongylognathus; taxonRank: Species; **Location:** country: Bulgaria; stateProvince: Haskovo; municipality: Ivaylovgrad; locality: Eastern Rhodopes, near Chernichino vill.; minimumElevationInMeters: 657; locationRemarks: in a nest of *Tetramorium hungaricum* Röszler, 1935; decimalLatitude: 41.5897; decimalLongitude: 25.8488; **Event:** eventDate: 03-05-2009; **Record Level:** collectionCode: BFUS; basisOfRecord: PreservedSpecimen
- **Type status:** Other material. **Occurrence:** recordedBy: A. Lapeva-Gjonova; individualCount: 1; sex: male; **Taxon:** scientificName: *Strongylognathus karawajewi*; order: Hymenoptera; family: Formicidae; genus: Strongylognathus; taxonRank: Species; **Location:** country: Bulgaria; stateProvince: Haskovo; municipality: Ivaylovgrad; locality: Eastern Rhodopes, near Chernichino vill.; minimumElevationInMeters: 657; locationRemarks: in a nest of *Tetramorium hungaricum* Röszler, 1935; decimalLatitude: 41.5897; decimalLongitude: 25.8488; **Event:** eventDate: 27-05-2012; **Record Level:** collectionCode: BFUS; basisOfRecord: PreservedSpecimen
- **Type status:** Other material. **Occurrence:** recordedBy: A. Lapeva-Gjonova; individualCount: 12; sex: workers; **Taxon:** scientificName: *Strongylognathus karawajewi*; order: Hymenoptera; family: Formicidae; genus: Strongylognathus; taxonRank: Species; **Location:** country: Bulgaria; stateProvince: Haskovo; municipality: Ivaylovgrad; locality: Eastern Rhodopes, near Chernichino vill.; minimumElevationInMeters: 657; locationRemarks: in a nest of *Tetramorium hungaricum* Röszler, 1935; decimalLatitude: 41.5897; decimalLongitude: 25.8488; **Event:** eventDate: 27-05-2012; **Record Level:** collectionCode: BFUS; basisOfRecord: PreservedSpecimen
- **Type status:** Other material. **Occurrence:** recordedBy: A. Lapeva-Gjonova; individualCount: 4; sex: workers; **Taxon:** scientificName: *Strongylognathus karawajewi*; order: Hymenoptera; family: Formicidae; genus: Strongylognathus; taxonRank: Species; **Location:** country: Bulgaria; stateProvince: Haskovo; municipality: Ivaylovgrad; locality: Eastern Rhodopes, near Chernichino vill.; minimumElevationInMeters: 657; locationRemarks: in a nest of *Tetramorium chefketi* Forel, 1911; decimalLatitude: 41.5897; decimalLongitude: 25.8488; **Event:** eventDate: 27-05-2012; **Record Level:** collectionCode: BFUS; basisOfRecord: PreservedSpecimen
- **Type status:** Other material. **Occurrence:** recordedBy: A. Lapeva-Gjonova; individualCount: 13; sex: workers; **Taxon:** scientificName: *Strongylognathus karawajewi*; order: Hymenoptera; family: Formicidae; genus: Strongylognathus; taxonRank: Species; **Location:** country: Bulgaria; stateProvince: Haskovo; municipality: Ivaylovgrad; locality: Eastern Rhodopes, near Chernichino vill.; minimumElevationInMeters: 657; locationRemarks: in a nest of *Tetramorium hungaricum* Röszler, 1935; decimalLatitude: 41.5897; decimalLongitude: 25.8488; **Event:** eventDate: 07-04-2013; **Record Level:** collectionCode: BFUS; basisOfRecord: PreservedSpecimen
- **Type status:** Other material. **Occurrence:** recordedBy: A. Lapeva-Gjonova; individualCount: 7; sex: workers; **Taxon:** scientificName: *Strongylognathus karawajewi*; order: Hymenoptera; family: Formicidae; genus: Strongylognathus; taxonRank: Species; **Location:** country: Bulgaria; stateProvince: Haskovo; municipality: Ivaylovgrad; locality: Eastern Rhodopes, near Chernichino vill.; minimumElevationInMeters: 657; locationRemarks: in a nest of *Tetramorium hungaricum* Röszler, 1935; decimalLatitude: 41.5897; decimalLongitude: 25.8488; **Event:** eventDate: 01-05-2019; **Record Level:** collectionCode: BFUS; basisOfRecord: PreservedSpecimen
- **Type status:** Other material. **Occurrence:** recordedBy: A. Lapeva-Gjonova; individualCount: 12; sex: workers; **Taxon:** scientificName: *Strongylognathus karawajewi*; order: Hymenoptera; family: Formicidae; genus: Strongylognathus; taxonRank: Species; **Location:** country: Bulgaria; stateProvince: Haskovo; municipality: Madzharovo; locality: Eastern Rhodopes, Gaberovo vill.; minimumElevationInMeters: 535; locationRemarks: in a nest of *Tetramorium chefketi* Forel, 1911 together with queens of the host; decimalLatitude: 41.6297; decimalLongitude: 25.8940; **Event:** eventDate: 24-04-2012; **Record Level:** collectionCode: BFUS; basisOfRecord: PreservedSpecimen
- **Type status:** Other material. **Occurrence:** recordedBy: A. Lapeva-Gjonova; individualCount: 1; sex: worker; **Taxon:** scientificName: *Strongylognathus karawajewi*; order: Hymenoptera; family: Formicidae; genus: Strongylognathus; taxonRank: Species; **Location:** country: Bulgaria; stateProvince: Kardzhali; municipality: Krumovgrad; locality: Eastern Rhodopes, Malko Kamenyane vill.; minimumElevationInMeters: 236; decimalLatitude: 41.4112; decimalLongitude: 25.6601; **Event:** eventDate: 29-04-2019; **Record Level:** collectionCode: BFUS; basisOfRecord: PreservedSpecimen

#### Description

SEM images: Fig. 3

#### Conservation

Vulnerable D2 ver. 2.3 (IUCN 2021)

#### Taxon discussion

*Strongylognathus karawajewi* was described, based on workers from the southern coast of Crimea (vill. Magarach, now Otradnoye near Yalta, material from the collection of W. A. Karawajew, ZISK) by Pisarski (1966). Later, it was recorded from the western Caucasus (Arnoldi and Dlussky 1978), the Kopetdagh Mts. (Dlussky and Zabelin 1985, Dlussky et al. 1990), Armenia and Hissar Range in Tadzhikistan (Radchenko 1991) and Turkey (Kiran et al. 2014); records of this species from China were based on misidentifications (Radchenko et al. 2017). Queens of *S. karawajewi* were described by Radchenko (1985) from Crimea and males – from Crimea, Armenia and Tajikistan (Radchenko 1991).

*Strongylognathus karawajewi* has been known as a social parasite of *T. caespitum* (s. l.), *T. sulcinode* Santschi, 1927, *T. inerme* Mayr, 1877, *T. ferox* Ruzsky, 1903 and *T. feroxoide* Dlussky & Zabelin, 1985 (Pisarski 1966, Radchenko 1991, Radchenko 2016) and we found it in the nests of *T. hungaricum* and *T. chefketi*.

This species inhabits extremely xerothermic sites in Bulgaria at an altitude below 660 m. One of them is located in the Besaparski Hills in the Thracian plain - low calcareous ridges with typical steppe-like vegetation (Fig. 1a), one in the southern foothills of the Stara Planina Mts. (Fig. 1b) and five of them in the Eastern Rhodopes (Fig. 1c). The Eastern Rhodopes are quite low with an average altitude of 320 m and with hilly slopes. The climate in this area is mild continental-Mediterranean with an average annual temperature 12°C.

Two species of the *testaceus* species-group (*S. testaceus* and *S. karawajewi*) are known from the West Palaearctic and their separation is usually straightforward. The head dorsum in workers of *S. karawajewi* is usually completely smooth and shiny, fine striation may be present only on its sides, while at least frons and genae, but often whole head dorsum, is with well developed longitudinal rugosity in *S. testaceus*. The sculpture on the sides of mesosoma in males of *S. karawajewi* is strongly reduced, but it is at least partly coarsely rugulose and shagreened in *S. testaceus* (Radchenko 1991, Radchenko 2016).

### Strongylognathus bulgaricus

Pisarski, 1966, stat. rev.

7BCCBF71-92BC-5DA7-9192-A6716789F59F

Strongylognathus huberi subsp. rehbinderi var. bulgarica Viehmeyer, 1922: 211, w, q, m, Bulgaria (unavailable name).
Strongylognathus rehbinderi subsp. bulgaricus : Pisarski 1966: 515 (first available use of name).
Strongylognathus bulgaricus : Bolton 1976: 306; Atanassov and Dlussky 1992: 158.
Strongylognathus bulgaricus *Strongylognathus kratochvili*Pisarski 1966*bulgaricuskratochvili*
S. bulgaricus is revived from synonymy with *S. kratochvili* and synonymised with *Strongylognathus christophi* Emery, 1889: 439, q, Russia: Seifert 2018: 237 (latter not confirmed here).
Strongylognathus huberi subsp. rehbinderi var. bulgarica Viehmeyer, 1922: 211, w, q, m, Bulgaria (unavailable name).
Strongylognathus rehbinderi subsp. bulgaricus : Pisarski 1966: 515 (first available use of name).
Strongylognathus bulgaricus : Bolton 1976: 306; Atanassov and Dlussky 1992: 158.
Senior synonym of *Strongylognathus kratochvili* Šilhavý, 1937: 5, w, q, Czech Republic: Pisarski 1966: 515 (*bulgaricus* given as senior synonym, but *kratochvili* has priority).
S. bulgaricus is revived from synonymy with *S. kratochvili* and synonymised with Strongylognathus christophi Emery, 1889: 439, q, Russia: Seifert 2018: 237 (latter not confirmed here).

#### Materials

- **Type status:** Other material. **Occurrence:** recordedBy: P. Bezdecka; individualCount: 2; sex: workers; **Taxon:** scientificName: *Strongylognathus bulgaricus*; order: Hymenoptera; family: Formicidae; genus: Strongylognathus; taxonRank: Species; **Location:** country: Bulgaria; locality: Kardzhali; **Event:** startDayOfYear: 24; endDayOfYear: 26; year: 1986; month: 04; **Record Level:** collectionCode: P. Werner; basisOfRecord: PreservedSpecimen
- **Type status:** Other material. **Occurrence:** recordedBy: A. Lapeva-Gjonova; individualCount: 1; sex: worker; **Taxon:** scientificName: *Strongylognathus bulgaricus*; order: Hymenoptera; family: Formicidae; genus: Strongylognathus; taxonRank: Species; **Location:** country: Bulgaria; stateProvince: Burgas; municipality: Tsarevo; locality: Southern Black Sea coast; minimumElevationInMeters: 5; decimalLatitude: 42.0233; decimalLongitude: 28.0083; **Event:** eventDate: 09-05-2009; **Record Level:** collectionCode: BFUS; basisOfRecord: PreservedSpecimen
- **Type status:** Other material. **Occurrence:** recordedBy: A. Lapeva-Gjonova; individualCount: 19; sex: workers; **Taxon:** scientificName: *Strongylognathus bulgaricus*; order: Hymenoptera; family: Formicidae; genus: Strongylognathus; taxonRank: Species; **Location:** country: Bulgaria; stateProvince: Kardzhali; municipality: Krumovgrad; locality: Eastern Rhodopes, near Dolna Kula vill.; minimumElevationInMeters: 257; locationRemarks: xerothermic grassland; decimalLatitude: 41.5583; decimalLongitude: 25.6414; **Event:** eventDate: 19-04-2012; **Record Level:** collectionCode: BFUS; basisOfRecord: PreservedSpecimen

#### Description

SEM images: Fig. 4

#### Conservation

Vulnerable D2 ver. 2.3 (IUCN 2021)

#### Taxon discussion

The previous records of the species in Bulgaria are from northern Bulgaria – Veliko Tarnovo, Preobrazhenski Monastery (10 km from Veliko Tarnovo), Dryanovo, Veliki Preslav and one (Silistar) is on the southern Black Sea coast (Viehmeyer 1922, Atanassov and Dlussky 1992, Lapeva-Gjonova and Kiran 2012). All northern Bulgarian sites are located in the Predbalkan geographic region, in a hilly and lowland area with an average altitude of 360 m.

Viehmeyer (1922) described S. huberi subsp. rehbinderi var. bulgaricus, based on all three castes from Veliko Tarnovo (northern Bulgaria), but this name is unavailable (quadrinomen). Pisarski (1966) used the first available name for this species, S. rehbinderi subsp. bulgaricus and considered it as a senior synonym of *S. kratochvili* Šilhavý, 1937, but the latter name has priority. It was later recorded for the country under the name *S. bulgaricus* by Atanassov and Dlussky (1992) and under the name *S. kratochvili* by Lapeva-Gjonova et al. (2010).

Recently, Seifert (2018) noted, without comments, that *S. bulgaricus* is not a synonym of *S. kratochvili*, but is a junior synonym of *S. christophi*. In our opinion, the proposed synonymy seems doubtful: the sculpture on the head dorsum in *S. christophi* is much coarser, the head is relatively shorter (CI = 1.10), the antennal scape is longer (SI > 0.70), the petiolar node with widely rounded dorsum and the propodeal dents are directed mostly backwards at an angle of ca. 45^o^. On the contrary, the sculpture on the head dorsum in *S. bulgaricus* is strongly reduced, its head is relatively longer (CI > 1.16), the antennal scape is shorter (SI < 0.70), the petiolar node with much more narrowly rounded dorsum and the propodeal dents are directed almost upwards (Table 1 and our unpublished data; see also Forel 1900, Šilhavý 1937).

On the other hand, *S. bulgaricus* and *S. kratochvili* are very similar to each other in many subjective features (e.g. sculpture of the head and mesosoma, pilosity, shape of the propodeal dents etc.), but *S. kratochvili* differs from *S. bulgaricus* by noticeably larger body size (it is one of the largest *Strongylognathus* species, as Šilhavý has already emphasised). We agree with the proposed separation of these species (see Seifert 2018), but consider *S. bulgaricus* (at least tentatively) a good species.

### Strongylognathus huberi dalmaticus

Baroni Urbani, 1969

6DCD478E-0E4D-5E26-A02D-4C9E7B774BB5

Strongylognathus dalmaticus Baroni Urbani, 1969: 154, w, Croatia; Agosti and Collingwood 1987: 278; Collingwood 1993: 195; Legakis 2011: 20; Borowiec and Salata 2012: 536.
Strongylognathus huberi dalmaticus : Borowiec and Salata 2013: 365; Salata and Borowiec 2018: 63; Salata et al. 2020: 15, 63.
Strongylognathus dalmaticus Baroni Urbani, 1969: 154, w, Croatia; Agosti and Collingwood 1987: 278; Collingwood 1993: 195; Legakis 2011: 20; Borowiec and Salata 2012: 536.
Strongylognathus huberi dalmaticus : Borowiec and Salata 2013: 365; Salata and Borowiec 2018: 63; Salata et al. 2020: 15, 63.

#### Materials

- **Type status:** Other material. **Occurrence:** recordedBy: A. Lapeva-Gjonova; individualCount: 15; sex: workers; **Taxon:** scientificName: *Strongylognathus huberi dalmaticus*; order: Hymenoptera; family: Formicidae; genus: Strongylognathus; taxonRank: subspecies; **Location:** country: Bulgaria; stateProvince: Haskovo; municipality: Ivaylovgrad; locality: Eastern Rhodopes, near Meden buk vill.; minimumElevationInMeters: 124; locationRemarks: in a nest of *Tetramorium hungaricum* Röszler, 1935; decimalLatitude: 41.3822; decimalLongitude: 26.017; **Event:** eventDate: 09-04-2013; **Record Level:** collectionID: BFUS; basisOfRecord: PreservedSpecimen
- **Type status:** Other material. **Occurrence:** recordedBy: A. Lapeva-Gjonova; individualCount: 2; sex: workers; **Taxon:** scientificName: *Strongylognathus huberi dalmaticus*; order: Hymenoptera; family: Formicidae; genus: Strongylognathus; taxonRank: subspecies; **Location:** country: Bulgaria; stateProvince: Haskovo; municipality: Madzharovo; locality: Eastern Rhodopes, near Senoklas vill.; minimumElevationInMeters: 285; locationRemarks: in a nest of *Tetramorium hungaricum* Röszler, 1935; decimalLatitude: 41.6066; decimalLongitude: 25.9394; **Event:** eventDate: 21-04-2014; **Record Level:** collectionCode: BFUS; basisOfRecord: PreservedSpecimen

#### Description

SEM images: Fig. 5

#### Conservation

Vulnerable D2 ver. 2.3 (IUCN 2021)

#### Taxon discussion

*Strongylognathus dalmaticus* was described by Baroni Urbani (1969) from Biševo Island (the Dalmatian Archipelago, Croatia) and later was also recorded from Greece (Collingwood 1993, Legakis 2011, Borowiec and Salata 2012, Borowiec and Salata 2013, Borowiec and Salata 2017), Crete (Borowiec and Salata 2013, Salata et al. 2020) and Bosnia and Herzegovina (Vesnic 2013). Till recently, it has been considered as a good species, but Borowiec and Salata (2013) and Salata et al. (2020) proposed to consider *S. dalmaticus* as a subspecies of *S. huberi* Forel, 1874.

It is no coincidence that this species is found in the Eastern Rhodopes, where the influence of the warmer Mediterranean climate is stronger and xerothermic plant communities are present. The collecting site near the village of Meden Buk is located in the valley of the Byala Reka River near the Greek border and it is one of the southernmost points of Bulgaria.

### Strongylognathus huberi dalmaticus

Baroni Urbani, 1969

5519CAC3-6BDA-544E-B684-AAA3EDA19B3A

Strongylognathus dalmaticus Baroni Urbani, 1969: 154, w, Croatia; Agosti and Collingwood 1987: 278; Collingwood 1993: 195; Legakis 2011: 20; Borowiec and Salata 2012: 536.
Strongylognathus huberi dalmaticus : Borowiec and Salata 2013: 365; Salata and Borowiec 2018: 63; Salata et al. 2020: 15, 63.
Strongylognathus dalmaticus Baroni Urbani, 1969: 154, w, Croatia; Agosti and Collingwood 1987: 278; Collingwood 1993: 195; Legakis 2011: 20; Borowiec and Salata 2012: 536.
Strongylognathus huberi dalmaticus : Borowiec and Salata 2013: 365; Salata and Borowiec 2018: 63; Salata et al. 2020: 15, 63.

#### Description

SEM images: Fig. 5

#### Conservation

Vulnerable D2 ver. 2.3 (IUCN 2021)

#### Taxon discussion

*Strongylognathus dalmaticus* was described by Baroni Urbani (1969) from Biševo Island (the Dalmatian Archipelago, Croatia) and later was also recorded from Greece (Collingwood 1993, Legakis 2011, Borowiec and Salata 2012, Borowiec and Salata 2013, Borowiec and Salata 2017), Crete (Borowiec and Salata 2013, Salata et al. 2020) and Bosnia and Herzegovina (Vesnic 2013). Till recently, it has been considered as a good species, but Borowiec and Salata (2013) and Salata et al. (2020) proposed to consider *S. dalmaticus* as a subspecies of *S. huberi* Forel, 1874.

It is no coincidence that this species is found in the Eastern Rhodopes, where the influence of the warmer Mediterranean climate is stronger and xerothermic plant communities are present. The collecting site near the village of Meden Buk is located in the valley of the Byala Reka River near the Greek border and it is one of the southernmost points of Bulgaria.

### Strongylognathus italicus

Finzi, 1924

FCD4907B-D30F-583D-BE3C-16194D537DEF

Strongylognathus huberi subsp. italica Finzi, 1924a: 14, q, Italy (Ils. Elba); Baroni Urbani 1971: 149.
Strongylognathus italicus : Bolton 1976: 308; Sanetra et al. 1999: 348; Borowiec 2014: 164.
Strongylognathus italicus *Strongylognathus alboini*[Bibr B6777925][Bibr B6778141]
Strongylognathus huberi subsp. italica Finzi, 1924a: 14, q, Italy (Ils. Elba); Baroni Urbani 1971: 149.
Strongylognathus italicus : Bolton 1976: 308; Sanetra et al. 1999: 348; Borowiec 2014: 164.
As senior synonym of *Strongylognathus alboini*Finzi 1924b: 121, w, Slovenia: Seifert 2018: 239.

#### Materials

- **Type status:** Other material. **Occurrence:** recordedBy: A. Lapeva-Gjonova; individualCount: 25; sex: workers; **Taxon:** scientificName: *Strongylognathus italicus*; order: Hymenoptera; family: Formicidae; genus: Strongylognathus; taxonRank: Species; **Location:** country: Bulgaria; stateProvince: Haskovo; municipality: Ivaylovgrad; locality: Eastern Rhodopes, near Chernichino vill.; minimumElevationInMeters: 657; locationRemarks: xerothermic grassland, in a nest of *Tetramorium chefketi* Forel, 1911; decimalLatitude: 41.5897; decimalLongitude: 25.8488; **Event:** eventDate: 07-04-2013; **Record Level:** collectionCode: BFUS; basisOfRecord: PreservedSpecimen

#### Description

SEM images: Fig. 6

#### Conservation

Vulnerable D2 ver. 2.3 (IUCN 2021)

#### Taxon discussion

Finzi (1924a) described *S. italicus*, based on a single queen from the Island of Elba and then Sanetra et al. (1999) recorded it from the same Island and from Italian mainland (Florence Province), but unfortunately, they did not indicate whether workers of this species were found.

In the same year, Finzi (1924b) described *S. alboini*, based on workers from Mt. Nanos (now Slovenia) and later, Baroni Urbani (1969) re-described its workers and described queens and males, based on the material collected by Kutter in southern Switzerland (Roveredo, Canton Cicino). Recently, Seifert (2018) compared morphometrically and subjectively the holotype queen of *S. italicus* with the queens of *S. alboinii* from Roveredo and concluded that they belong to the same species; in addition, workers of *S. italicus* from Roveredo are identical to syntype workers of *S. alboini* from Mt. Nanos. Consequently, he considered *S. alboinii* as junior synonym of *S. italicus*.

*Strongylognathus italicus* differs from other Bulgarian species of the *huberi*-group by the coarser sculpture on the head dorsum and somewhat longer antennal scape. In Bulgaria, it was found only once on a southern slope of xerothermic grassland situated in an oak forest (Fig. 1c) at an altitude of ca. 650 m in a nest of *Tetramorium chefketi*. It is interesting to note that, in the same site, we found *S. karawajewi* and the very rare social parasite of *Tetramorium* – *Teleutomyrmex buschingeri* Lapeva-Gjonova, 2017.

### Strongylognathus afer

Emery, 1884

0154372D-4CBF-5994-9EA1-CA842E4DE4CD

Strongylognathus afer Emery, 1884: 380, q, Algeria; Forel 1900: 279, w (in Key); Santschi 1910: 71, m; Emery 1909: 711; all subsequent authors.
Strongylognathus afer Emery, 1884: 380, q, Algeria; Forel 1900: 279, w (in Key); Santschi 1910: 71, m; Emery 1909: 711; all subsequent authors.

#### Materials

- **Type status:** Other material. **Occurrence:** recordedBy: A. Lapeva-Gjonova; individualCount: 19; sex: workers; **Taxon:** scientificName: *Strongylognathus afer*; order: Hymenoptera; family: Formicidae; genus: Strongylognathus; taxonRank: Species; **Location:** country: Bulgaria; stateProvince: Haskovo; municipality: Madzharovo; locality: Eastern Rhodopes, Gaberovo vill.; minimumElevationInMeters: 535; locationRemarks: on the border of light oak forest, in a nest of *Tetramorium hungaricum* Röszler, 1935; decimalLatitude: 41.6297; decimalLongitude: 25.8940; **Event:** eventDate: 10-04-2013; **Record Level:** collectionCode: BFUS; basisOfRecord: PreservedSpecimen

#### Description

SEM images: Fig. 7

#### Conservation

Vulnerable D2 ver. 2.3 (IUCN 2021)

#### Taxon discussion

*Strongylognathus afer* was described by Emery (1884), based on a single queen from Algeria, workers and males being later described from Algeria and Tunisia by Forel (1900) and Santschi (1910), respectively; finally, Sanetra and Güsten (2001) recorded this species in many localities in Algeria, Tunisia and Morocco. *Strongylognathus afer*, in all studied sites, infested colonies of *Tetramorium semilaeve* Andre, 1883 (Sanetra and Güsten 2001). Workers of this species are very small, the propodeum bearing small and blunt tubercles instead of sharp dents and the head dorsum and mesosoma are generally smooth and shiny (Forel 1900, Sanetra and Güsten 2001).

**Figure 4.**
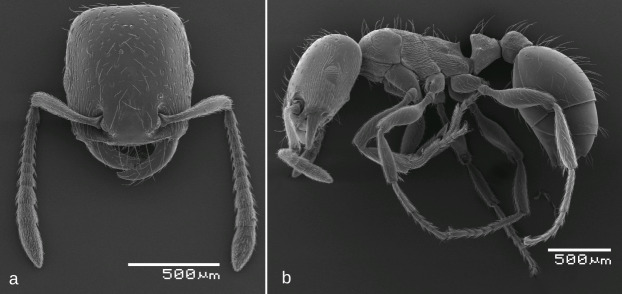
*Strongylognathus bulgaricus* Pisarski, 1966, workers: a – head, dorsal view; b – body, lateral view.

One nest sample of workers, together with the host species *T. hungaricum*, was collected in the Eastern Rhodopes on the border of light oak forest and a pasture with a southern exposure (Fig. 4) at an altitude about 550 m. Collected workers morphologically fit well with the main characteristic features of *S. afer*, but are even smaller than the specimens from Algeria and Morocco, as well as workers of *S. minutus* Radchenko, 1991 and, apparently, are the smallest known workers of the *huberi* species-group (compare Table 1 and data in Radchenko 1991 and Sanetra and Güsten 2001).

## Identification Key

### Key for identification of *Strongylognathus* species of Bulgaria (workers)

**Table:** 

1	Occipital margin of head strongly concave (seen from above), posterio-lateral corners of head strongly prominent (seen from sides) (Fig. 2a and Fig. 3a)	2
–	Occipital margin of head straight or, at most, very shallowly concave (seen from above), posterio-lateral corners of head rounded and not prominent (seen from sides) (Fig. 4a, Fig. 5a, Fig. 6a and Fig. 7a)	3
2	Whole head dorsum usually smooth and shiny, fine striation may be present only on the sides of head dorsum (Fig. 3a)	*S. karawajewi*
–	At least frons and genae (often whole head dorsum) with well developed longitudinal rugosity (Fig. 2a)	*S . testaceus*
3	Propodeum without dents, at most with blunt tubercles (Fig. 7b). Head dorsum mostly smooth, only genae occasionally with fine longitudinal striation (Fig. 7a). Smaller: mean HL = 0.70, mean ML < 0.85	*S. afer*
–	Propodeum with at least small sharp dents (Fig. 4b, Fig. 5b and Fig. 6b). Only central part of head dorsum smooth, at least its lateral parts with longitudinal rugulosity (Fig. 4a, Fig. 5a and Fig. 6a). Larger: mean HL > 0.75, mean ML > 0.90	4
4	Head sculpture coarser, longitudinal rugulae on lateral parts of head dorsum curve inside posteriorly and surround occipital margin (Fig. 6a). Scape longer, SI > 0.70 (mean 0.73)	*S. italicus*
–	Head sculpture weaker, longitudinal rugulae on lateral parts of head dorsum do not curve inside posteriorly, occipital margin smooth (Fig. 4a, Fig. 5a). Scape shorter, SI < 0.70 (mean 0.66-0.67)	5
5	Propodeal dents directed almost upwards; petiolar node dorsum narrowly rounded (Fig. 4b). Somewhat smaller, mean HL 0.77, mean ML 0.93	*S. bulgaricus*
–	Propodeal spines directed upwards and backwards at an angle of ca. 45°; petiolar node dorsum widely rounded (Fig. 5b). Somewhat larger, mean HL 0.81, mean ML 1.00	*S. huberi dalmaticus*

## Discussion

The record of *S. karawajewi* in Bulgaria is quite consistent with the zoogeographic data and today represents the westernmost edge of the range of this East Tethyan species. At first glance, it may seem that the finds of the north-west African or west Mediterranean species (e.g. *S. afer* and *S. italicus*) in Bulgaria are unlikely, but this is not entirely true. As mentioned above, at present the taxonomic situation in the *huberi* species-group, especially in the West Palaearctic (i.e. west of Yenisei River and the Tien Shan Mts.; see Radchenko and Elmes 2010), is very complicated, often rather confusing and most of the problems have not yet been finally resolved and require thorough revision.

Although *S. afer* is formally recorded only from the north-western Africa and its relationships with three other Iberian and Italian species is not fully resolved, their conspecificity appears quite possible. Sanetra and Güsten (2001) have shown that the difference between the holotype queen of *S. caeciliae* Forel, 1897 from the Iberian Peninsula and Algerian queens of *S. afer* is the same as the difference observed between samples of the latter species collected in Algeria and Tunisia; Italian *S. destefanii* Emery, 1915 obviously differs from *S. afer* only by its somewhat larger size; finally, *S. insularis* Baroni Urbani, 1968 from Malta almost certainly should be a synonym of *S. afer* and/or *S. destefanii*. A somewhat similar situation applies to *S. italicus* as indicated by Seifert (2018).

Previously, Bolton (1976) on p. 305 wrote: “… many of the species-level names in the genus may merely be localized populations and I am convinced that further collections and study will reduce the number of species in *Strongylognathus* to a much lower figure”. We can agree with this opinion and many modern nominal species in the end may turn out to be only separate populations of widespread species, as, for example, *S. afer* or *S. italicus* (or its putative senior synonyms).

In addition, it should be emphasised that *Strongylognathus* fauna is very poorly understood in the former Yugoslavian countries and this territory appears a “blind spot” between Italy on the west and Bulgaria in the east. Thus (excluding *S. alboini* and *S. huberi dalmaticus* with the type localities in Slovenia and Croatia), only one more species, the common *S. testaceus*, was recorded from Serbia, Croatia and Slovenia (Petrov and Collingwood 1992, Bracko 2006, Bracko 2007, Vesnic 2013); similarly, four *Strongylognathus* species were previously recorded for Greece ([Bibr B6957327][Bibr B6778000], Borowiec and Salata 2012, Borowiec and Salata 2013), but this number was recently reduced to three (Salata and Borowiec 2018). Considering that about ten species are known now in Italy, six in Bulgaria, five in Ukraine (Radchenko 2016) and eight in Turkey (Kiran et al. 2014, Kiran and Karaman 2020), the number of Balkan *Strongylognathus* species is definitely underestimated and further research may close the “blind spot” between Italy and Bulgaria.

## Supplementary Material

XML Treatment for Strongylognathus testaceus

XML Treatment for Strongylognathus karawajewi

XML Treatment for Strongylognathus bulgaricus

XML Treatment for Strongylognathus huberi dalmaticus

XML Treatment for Strongylognathus huberi dalmaticus

XML Treatment for Strongylognathus italicus

XML Treatment for Strongylognathus afer

## Figures and Tables

**Figure 2.**
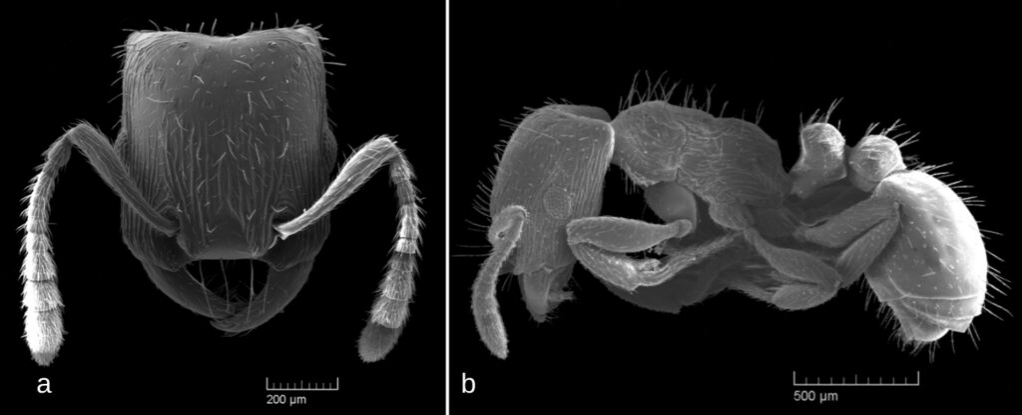
*Strongylognathus testaceus* (Schenck, 1852) workers: a – head, dorsal view; b – body, lateral view.

**Figure 3.**
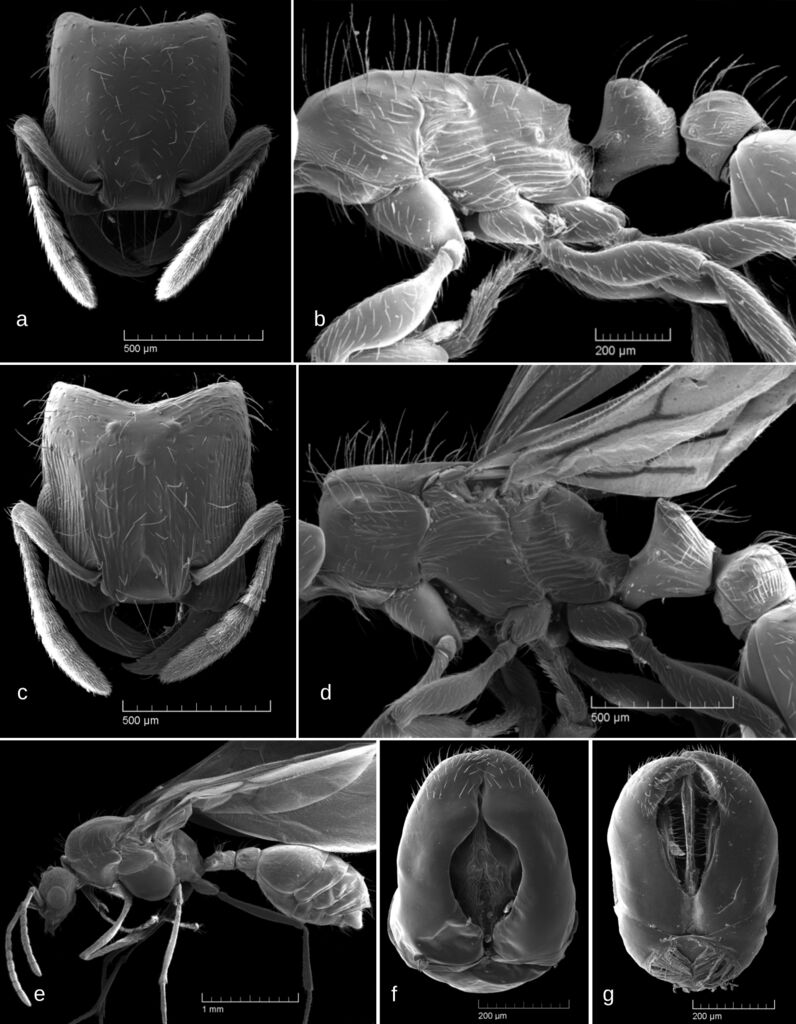
*Strongylognathus karawajewi* Pisarski, 1966: a, b – workers; c, d – gynes; e, f, g – males; a, c – head, dorsal view; b, d – mesosoma, lateral view; e – body, lateral view; f – male genitalia, stipites in dorsal view; g – male genitalia, stipites in ventral view.

**Figure 5.**
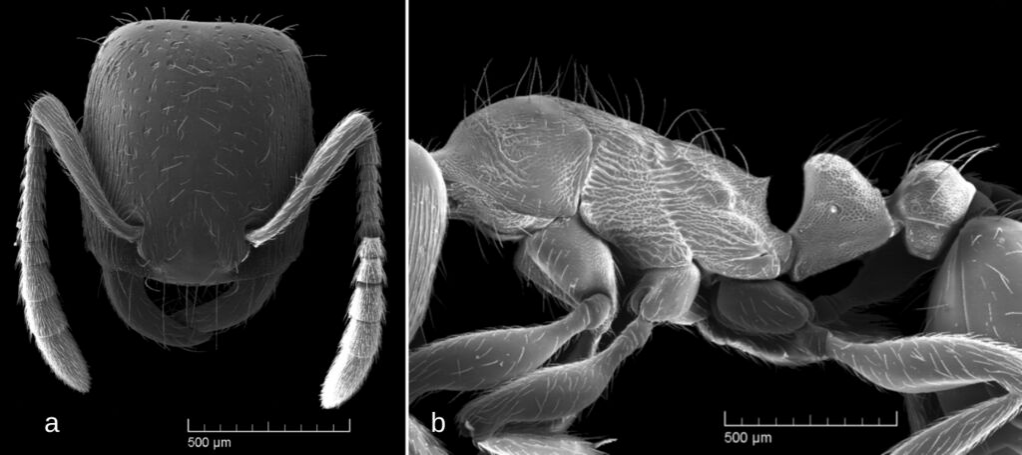
*Strongylognathus huberi dalmaticus* Baroni Urbani, 1969, workers: a – head, dorsal view; b – mesosoma lateral view.

**Figure 6.**
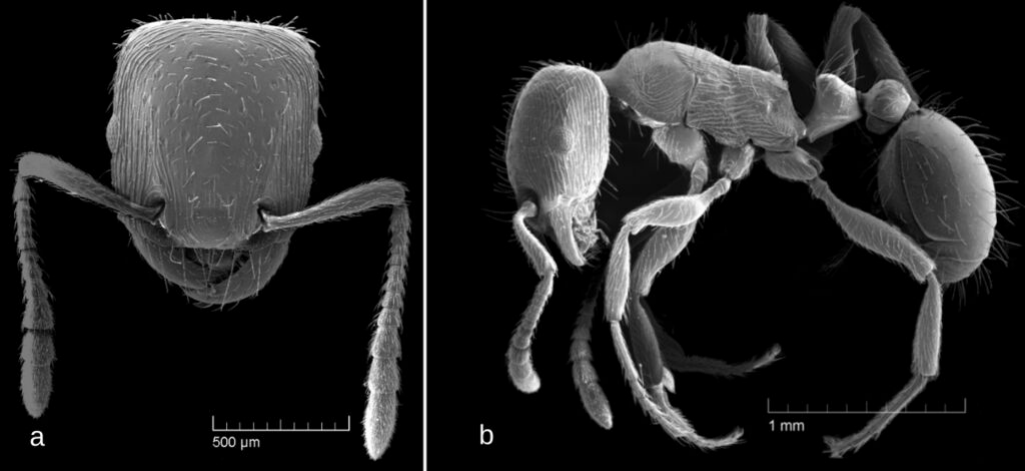
*Strongylognathus italicus* Finzi, 1924, workers: a – head, dorsal view; b – body, lateral view.

**Figure 7.**
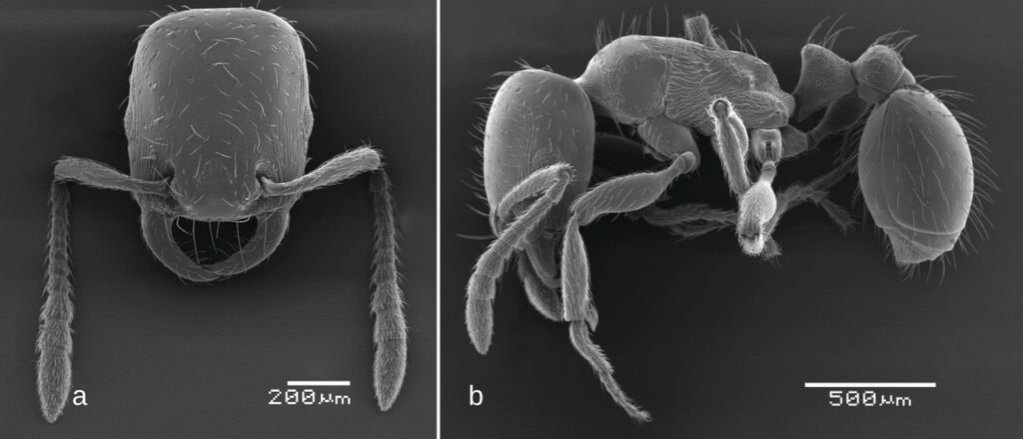
*Strongylognathus afer* Emery, 1884, workers: a. head, dorsal view; b. body, lateral view.

**Table 1.** Measurements (in mm) and indices of the investigated species of *Strongylognathus huberi*-group. Measured material: *S. bulgaricus*: Eastern Rhodopes, Dolna kula (17 workers); Kardzhali (2 workers); *S. huberi dalmaticus*: Eastern Rhodopes, Meden Buk; *S. italicus*: Eastern Rhodopes, near Chernichino vill.; *S. afer*: Eastern Rhodopes, Gaberovo vill.

	*S. bulgaricus* (n = 19)		*S. huberi dalmaticus* (n = 15)		*S. italicus* (n = 20)		*S. afer* (n = 17)	
	mean	min-max	mean	min-max	mean	min-max	mean	min-max
Measurements								
HL	0.774	0.719-0.825	0.807	0.754-0.842	0.811	0.754-0.912	0.693	0.632-0.719
HW	0.669	0.614-0.737	0.698	0.614-0.737	0.736	0.684-0.842	0.594	0.544-0.623
SL	0.511	0.474-0.544	0.543	0.509-0.561	0.587	0.544-0.649	0.468	0.421-0.491
ML	0.925	0.860-1.000	1.002	0.895-1.070	1.012	0.912-1.175	0.821	0.711-0.860
PW	0.200	0.175-0.237	0.228	0.193-0.246	0.227	0.193-0.263	0.179	0.158-0.193
PPW	0.270	0.246-0.298	0.299	0.263-0.333	0.306	0.281-0.351	0.227	0.211-0.246
Indices								
CI	1.157	1.119-1.200	1.158	1.095-1.228	1.101	1.069-1.129	1.166	1.141-1.206
SI	0.660	0.636-0.708	0.673	0.638-0.695	0.725	0.615-0.750	0.675	0.650-0.699
PI	0.742	0.687-0.843	0.761	0.722-0.801	0.741	0.685-0.795	0.788	0.749-0.846
PPI	0.403	0.381-0.425	0.428	0.405-0.452	0.416	0.395-0.445	0.382	0.354-0.400

## References

[B6777701] Agosti Donat, Collingwood Cedric A. (1987). A provisional list of the Balkan ants (Hym. Formicidae) with a key to the worker caste. II. Key to the worker caste, including the European species without the Iberian.. Mitteilungen der Schweizerischen Entomologischen Gesellschaft.

[B6777710] AntWeb AntWeb v 8.54.5. http://www.antweb.org/.

[B6777718] Arnoldi K. V., Dlussky G. M., Medvedev G. S. (1978). Superfamily Formicoidea. Family Formicidae. The ants.. Opredelitel' nasekomyh Evropejskoj chasti SSSR.

[B6777731] Atanassov Neno (1964). Studies on the systematics and ecology of ants (Formicidae, Hym.) from the Petrich region (SW Bulgaria).. Izvestiya na Zoologicheskiya Institut s Muzej. Bulgarska Akademiya na Naukite.

[B6777740] Atanassov N., Vasileva E., Peshev G. (1976). New and rare ant species (Hymenoptera, Formicidae) for the fauna of Bulgaria. Terrestrial fauna of Bulgaria. Materials..

[B6777753] Atanassov Neno, Dlussky G. M. (1992). Fauna Bulgarica. 22. Hymenoptera, Formicidae..

[B6777761] Baroni Urbani Cesare (1969). Gli *Strongylognathus* del gruppo *huberi* nell'Europa occidentale: saggio di una revisione basata sulla casta operaria. Bolletino della Società Entomologica Italiana.

[B6777770] Baroni Urbani Cesare (1971). Catalogo delle specie di Formicidae d'Italia. (Studi sulla mirmecofauna d'Italia - X). Memorie della Società Entomologica Italiana.

[B6777779] Bolton B. (1976). The ant tribe Tetramoriini (Hymenoptera: Formicidae). Constituent genera, review of smaller genera and revision of *Triglyphothrix* Forel. Bulletin of The British Museum (Natural History) Entomology.

[B6777788] Bolton B. An online catalog of the ants of the world. http://antcat.org.

[B6777796] Borowiec Lech, Salata Salata (2012). Ants of Greece – checklist, comments and new faunistic data (Hymenoptera: Formicidae). Genus.

[B6777805] Borowiec Lech, Salata Sebastian (2013). Ants of Greece – additions and corrections (Hymenoptera : Formicidae). Genus.

[B6777814] Borowiec L. (2014). Catalogue of ants of Europe, the Mediterranean Basin and adjacent regions (Hymenoptera: Formicidae). Genus.

[B6777823] Borowiec Lech, Salata Sebastian (2017). Ants of the Peloponnese, Greece (Hymenoptera: Formicidae). Polish Journal of Entomology.

[B6777832] Bracko Gregor (2006). Review of the ant fauna (Hymenoptera: Formicidae) of Croatia. Acta Entomologica Slovenica.

[B6777841] Bracko Gregor (2007). Checklist of the ants of Slovenia (Hymenoptera: Formicidae). Natura Sloveniae.

[B6957327] Buschinger A. (1989). Workerless *Epimyrma
kraussei* Emery, 1915, the first parasitic ant from Crete. Psyche.

[B6777850] Buschinger Alfred (2009). Social parasitism among ants: a review (Hymenoptera: Formicidae). Myrmecological News.

[B6777859] Collingwood C. A. (1993). A comparative study of the ant fauna of five Greek islands. Biologia Gallo-hellenica.

[B6777868] D'Ettorre Patrizia, Heinze Jürgen (2001). Sociobiology of slave-making ants. Acta Ethologica.

[B6777877] Dlussky G. M., Zabelin S. I., Nechaevaya N. T. (1985). The ant fauna (Hymenoptera, Formicidae) of the riv. Sumbar basin (south-western Kopetdag). Flora and fauna of the western Kopetdag..

[B6777890] Dlussky G. M., Sojunov O. S., Zabelin S. I. (1990). The ants of Turkmenistan.

[B6777898] Emery C. (1884). Materiali per lo studio della fauna Tunisina raccolti da G. e L. Doria. III. Rassegna delle formiche della Tunisia.. Annali del Museo Civico di Storia Naturale di Genova.

[B6777907] Emery C. (1909). Beiträge zur Monographie der Formiciden des paläarktischen Faunengebietes. (Hym.) IX.. Deutsche Entomologische Zeitschrift.

[B6777916] Finzi Bruno (1924). Formiche dell'isola d'Elba e Monte Argentario.. Bollettino della Società Entomologica Italiana.

[B6777925] Finzi B. (1924). Secondo contributo alla conoscenza della fauna mirmecologica della Venezia Giulia. Bollettino della Società Entomologica Italiana.

[B6777934] Forel Auguste (1900). Fourmis du Japon. Nids en toile. *Strongylognathus
huberi* et voisins. Fourmilière triple. *Cyphomyrmex
wheeleri*. Fourmis importées.. Mitteilungen der Schweizerischen Entomologischen Gesellschaft.

[B6777943] IUCN The IUCN Red List of Threatened Species. Version 2020-3. https://www.iucnredlist.org.

[B6777951] Kiran Kadri, Karaman Celal, Aksoy Volkan (2014). Atipic social parasitism among *Strongylognathus* (Hymenoptera, Formicidae)..

[B6777960] Kiran Kadri, Karaman Celal (2020). Additions to the ant fauna of Turkey (Hymenoptera, Formicidae). Zoosystema.

[B6777969] Lapeva-Gjonova Albena, Beron P., Popov A. (2004). Ants (Hymenoptera: Formicidae) from the Eastern Rhodopes (Bulgaria). Biodiversity of Bulgaria. 2. Biodiversity of Eastern Rhodopes (Bulgaria and Greece)..

[B6777982] Lapeva-Gjonova Albena, Antonova Vera, Radchenko A. G., Atanasova M. (2010). Catalogue of the ants (Hymenoptera: Formicidae) of Bulgaria. ZooKeys.

[B6777991] Lapeva-Gjonova Albena, Kiran Kadri (2012). Ant fauna (Hymenoptera, Formicidae) of Strandzha Mountain (Istranca) and adjacent Black Sea coast. North-western Journal of Zoology.

[B6778000] Legakis Anastasios (2011). Annotated list of the ants (Hymenoptera, Formicidae) of Greece. Hellenic Zoological Archives.

[B6778009] Mayr Gustav L. (1853). Ueber die Abtheilung der Myrmiciden, und eine neue Gattung derselben. Verhandlungen der Zoologisch-Botanischen Vereins in Wien.

[B6778018] Petrov I. Z., Collingwood C. A. (1992). Survey of the myrmecofauna (Formicidae, Hymenoptera) of Yugoslavia. Archives of Biological Science (Belgrade).

[B6778027] Pisarski Bohdan (1966). Études sur les fourmis du genre *Strongylognathus* Mayr (Hymenoptera, Formicidae). Annales Zoologici.

[B6778036] Radchenko Alexander (2005). Monographic revision of the ants (Hymenoptera: Formicidae) of North Korea. Annales Zoologici.

[B6778045] Radchenko Alexander G., Elmes Graham W. (2010). *Myrmica* ants (Hymenoptera: Formicidae) of the Old World.

[B6778053] Radchenko Alexander G., Zhang Yichen, Heinze Jürgen (2017). A new species of the ant genus *Strongylognathus* (Hymenoptera, Formicidae) from Inner Mongolia, with notes on the species reported from China. Asian Myrmecology.

[B6778062] Radchenko A. G. (1985). Ants of the genus *Strongylognathus* (Hymenoptera, Formicidae) in the European part of the USSR.. Zoologicheskii Zhurnal.

[B6778071] Radchenko A. G. (1991). Ants of genus *Strongylognathus* (Hymenoptera, Formicidae) from the USSR fauna. Zoologicheskii Zhurnal.

[B6778080] Radchenko A. G. (2016). The ants (Hymenoptera, Formicidae) of Ukraine.

[B6957177] Salata S., Borowiec L. (2018). Taxonomic and faunistic notes on Greek ants (Hymenoptera: Formicidae). Annals of the Upper Silesian Museum in Bytom Entomology.

[B6778088] Salata Sebastian, Borowiec Lech, Trichas Apostolos (2020). Review of ants (Hymenoptera: Formicidae) of Crete, with keys to species determination and zoogeographical remarks Formicidae.

[B6778096] Sanetra Matthias, Güsten Robert, Schulz Andreas (1999). On the taxonomy and distribution of Italian *Tetramorium* species and their social parasites (Hymenoptera
Formicidae). Memorie della Società Entomologica Italiana.

[B6778105] Sanetra Matthias, Buschinger Alfred (2000). Phylogenetic relationships among social parasites and their hosts in the ant tribe Tetramoriini (Hymenoptera: Formicidae). European Journal of Entomology.

[B6778114] Sanetra Matthias, Güsten Robert (2001). The socially parasitic ant genus *Strongylognathus* Mayr in North Africa (Insecta: Hymenoptera: Formicidae). Zootaxa.

[B6778123] Santschi F. (1910). Nouvelles Fourmis de Tunisie (suite). Bulletin de la Société d'Histoire Naturelle de l'Afrique du Nord.

[B6778132] Schulz Andreas, Sanetra Matthias (2002). Notes on the socially parasitic ants of Turkey and the synonymy of *Epimyrma* (Hymenoptera, Formicidae). Entomofauna.

[B6778141] Seifert Bernhard (2018). The Ants of the Central and North Europe.

[B6778149] Šilhavý Vladimir (1937). *Strongylognathus
kratochvili* n. sp., nový praeglacialni mravenec z Moravy. Sborník Prírodovedeckého Klubu v Trebíci.

[B6778158] Vesnic Adi (2013). The first record of the ant genus *Strongylognathus* (Insecta: Hymenoptera: Formicidae) in Bosnia and Herzegovina with notes on the distribution of the genus in the western part of the Balkan Peninsula. Acta Entomologica Serbica.

[B6778167] Viehmeyer H (1922). Neue Ameisen. Archiv für Naturgeschichte.

[B6778176] Wagner Herbert C., Arthofer Wolfgang, Seifert Bernhard, Muster Christoph, Steiner Florian M., Schlick-Steiner Birgit C. (2017). Light at the end of the tunnel: Integrative taxonomy delimits cryptic species in the *Tetramorium
caespitum* complex (Hymenoptera: Formicidae). Myrmecological News.

